# Long-Term Bergamot Essential Oil Inhalation Alleviates Prolonged Exercise-Induced Fatigue in Rats Through the Modulation of Skeletal Muscle Inflammation, Oxidative Stress, and Regeneration-Related Signaling

**DOI:** 10.3390/ijms27156736

**Published:** 2026-07-28

**Authors:** Lei Tian, Kaixin Shi, Siyi Pan

**Affiliations:** 1 College of Food Science and Technology, Huazhong Agricultural University, Wuhan 430070, China; leitian626@gmail.com; 2 Key Laboratory of Environment Correlative Dietology, Ministry of Education, Huazhong Agricultural University, Wuhan 430070, China; 3 Key Laboratory of Fruit & Vegetable Processing & Quality Control, Huazhong Agricultural University, Wuhan 430070, China; 4 Department of Cell Biology and Human Anatomy, School of Medicine, University of California at Davis, Davis, CA 95616, USA; 5 Agricultural Products Processing Research Institute, Chongqing Academy of Agricultural Science, Chongqing 401329, China; skx@webmail.hzau.edu.cn

**Keywords:** bergamot essential oil, exercise-induced fatigue, fatigue relief, peripheral fatigue, volatile bioactives

## Abstract

Excessive exercise without adequate recovery can induce prolonged fatigue, accompanied by skeletal muscle inflammation, oxidative stress, and impaired repair. Bergamot essential oil (BEO), a citrus-derived volatile essential oil, has been reported to have anti-inflammatory and antioxidant activities, but its effects on exercise-induced prolonged fatigue remain unclear. This study investigated the fatigue-relieving effects of long-term inhalation of BEO and its major constituents in a rat model of prolonged exercise-induced fatigue, with emphasis on skeletal muscle regulation. Rats were subjected to progressive exercise loading and exposed daily to BEO or its main components, followed by behavioral, biochemical, histological, transcriptional, and protein analyses. BEO inhalation significantly improved swimming endurance and alleviated fatigue-related physiological disturbances. In skeletal muscle, BEO reduced IL-1β and IL-6 levels, attenuated transcriptional changes in NF-κB/NLRP3-related inflammatory markers, decreased MDA accumulation, and enhanced GSH-Px activity. BEO also modulated muscle repair-related markers by reversing the abnormal elevation of Myod1 and restoring Myog mRNA expression. These effects were accompanied by increased Igf1 expression, restored PI3K protein expression, enhanced PI3K phosphorylation, and increased inhibitory phosphorylation of GSK3β. Overall, BEO alleviated prolonged exercise-induced fatigue by attenuating skeletal muscle inflammation, oxidative stress, and repair-related dysregulation, supporting its potential as a non-oral aromatic intervention for fatigue recovery.

## 1. Introduction

Physical fatigue is a complex and multifaceted phenomenon characterized by physical and/or mental exhaustion resulting from high-intensity exercise, chronic stress, or prolonged mental activity [1]. Persistent strenuous exercise, stress, or mental work, coupled with inadequate recovery, can lead to a prolonged fatigue state. The imbalance between fatigue and recovery can lead to declines in physical performance and various physiological and biochemical impairments. Unlike acute fatigue, which is usually reversible after short-term rest, prolonged exercise-induced fatigue reflects a sustained imbalance between physical load and recovery capacity. Skeletal muscle is a primary peripheral tissue responsible for force generation, energy consumption, and post-exercise recovery [2]. Under excessive or prolonged exercise stress, skeletal muscle may undergo energy depletion, oxidative imbalance, inflammatory activation, and structural injury, which together contribute to reduced endurance performance and delayed recovery [1].

Over the past decades, numerous nutrients and natural active ingredients in herbs and foods, such as BCAAs, vitamins, curcumin, and ginseng, have been found to help alleviate fatigue [3]. However, natural volatile compounds have attracted increasing attention as non-invasive interventions for fatigue management since they can be administered via inhalation and may influence both physiological and psychological dimensions of fatigue [4,5]. Previous studies have reported that inhalation of essential oils can improve exercise performance and reduce fatigue-related biochemical disturbances, suggesting a potential role of aromatic intervention in exercise recovery [6,7]. In skeletal muscle, fatigue recovery is closely associated with the restoration of redox homeostasis, resolution of inflammatory responses, and activation of repair-related signaling [8]. The PI3K/AKT pathway and its downstream targets, including GSK3β and mTOR, are involved in metabolic regulation, protein synthesis, and muscle repair, and therefore may represent important molecular indicators of skeletal muscle recovery under prolonged exercise stress [9].

Citrus essential oils (CEOs), by-products of citrus processing, are generally recognized as safe (GRAS) and are widely applied in functional foods and aromatherapy [10]. Due to their high volatility and lipophilicity, essential oils can be efficiently delivered through inhalation, allowing volatile compounds to interact with the olfactory and respiratory systems and may subsequently modulate systemic physiological responses [11,12]. Bergamot essential oil (Citrus bergamia Risso et Poiteau, BEO), which is rich in linalyl acetate, D-limonene, and linalool, showed promising fatigue-relieving activity in our previous work [7]. However, whether long-term BEO inhalation promotes peripheral muscle recovery under prolonged exercise-induced fatigue, and whether its major volatile components contribute similarly to this effect, remains unclear.

Therefore, the present study aimed to investigate the effects of long-term BEO inhalation and its main components on prolonged exercise-induced fatigue (prolonged fatigue for brevity), with a particular focus on skeletal muscle oxidative stress, inflammatory responses, muscle injury-repair balance, and PI3K/AKT/GSK3β/mTOR-related signaling.

## 2. Results

### 2.1. Behavior Performance and Organ Coefficient

**Figure 1:**
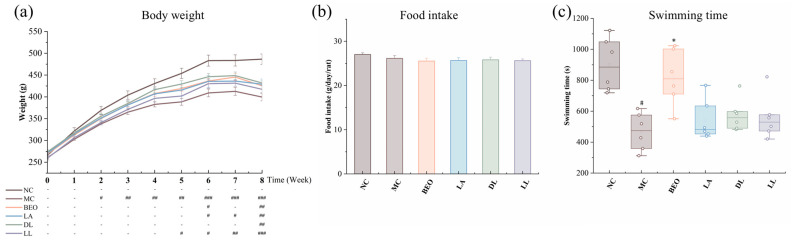
Effect of BEO and its main components on (**a**) body weight, (**b**) food intake, (**c**) swimming ability. Values given are means ± SEMs, with *n* = 6. ^#^ *p* < 0.05, ^##^ *p* < 0.01 or ^###^ *p* < 0.001 indicates that the difference is significant compared with the NC group; * *p* < 0.05 indicates that the difference is significant compared with the MC group.

After 8 weeks of exercise, the weight of the five exercising groups except the NC group significantly decreased by 18%, 13%, 12%, 11%, and 14%, respectively, compared to the NC group (*p* < 0.001, Figure 1a), confirming that physiological stress was induced by the protocol. However, no significant differences were observed among the MC, BEO, LA, DL, and LL groups (*p* > 0.05). Food intake remained stable across the groups (*p* > 0.05, Figure 1b). Notably, the swimming time of the MC group decreased significantly, as shown in Figure 1c (*p* < 0.05), indicating the successful establishment of a prolonged exercise-induced fatigue model. BEO administration significantly prolonged swimming duration compared with the MC group (*p* < 0.05; Figure 1c), whereas LA, DL, and LL showed modest but non-significant improvement trends. As shown in Table 1, the organ coefficients of the spleen and lung were not significantly different among the groups. However, the liver index was significantly increased in the BEO group compared with the NC and MC groups (*p* < 0.05), whereas no significant increase was observed in the monomer groups (*p* > 0.05). Together, these results indicated that BEO may be effective in alleviating prolonged fatigue.

### 2.2. H&E Staining of Skeletal Muscle

**Figure 2:**
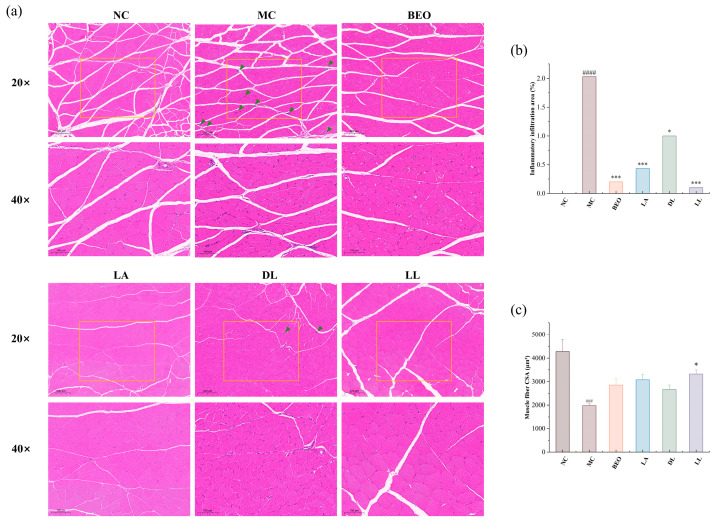
Effect of BEO and its main components on (**a**) skeletal muscle pathologies (20.0× and 40.0×), (**b**) percentage of inflammatory infiltration area, and (**c**) muscle fiber CSA. ^###^ *p* < 0.001, ^####^ *p* < 0.0001, indicates that the difference is significant compared with the NC group; * *p* < 0.05 or *** *p* < 0.001 indicates a significant difference compared with the MC group. The orange box in the 20× image indicates the region shown at higher magnification (40×).

As shown in Figure 2, H&E staining revealed generally intact skeletal muscle fiber morphology across groups, without obvious disruption of fiber arrangement. However, compared with the NC group, the MC group displayed increased inflammatory cell accumulation in the interstitial regions, along with reduced muscle fiber size. Quantitative analysis of H&E-stained skeletal muscle sections showed that the inflammatory infiltration area was markedly increased in the MC group compared with the NC group, confirming the presence of exercise-induced inflammatory cell accumulation in skeletal muscle. BEO treatment significantly reduced the inflammatory infiltration area, and similar decreases were observed in the LA, DL, and LL groups, indicating that BEO and its major constituents alleviated inflammatory cell infiltration to varying degrees. In addition, muscle fiber cross-sectional area (CSA) was significantly decreased in the MC group compared with the NC group, suggesting atrophy-like morphological alterations after prolonged exercise-induced fatigue. Compared with the MC group, the LL group showed significantly increased muscle fiber CSA. Although the changes in the BEO, LA, and DL groups did not reach statistical significance, these groups showed an increasing trend in CSA, suggesting partial improvement of muscle fiber morphology.

### 2.3. Inflammation Cytokine Levels in Skeletal Muscle

**Figure 3:**
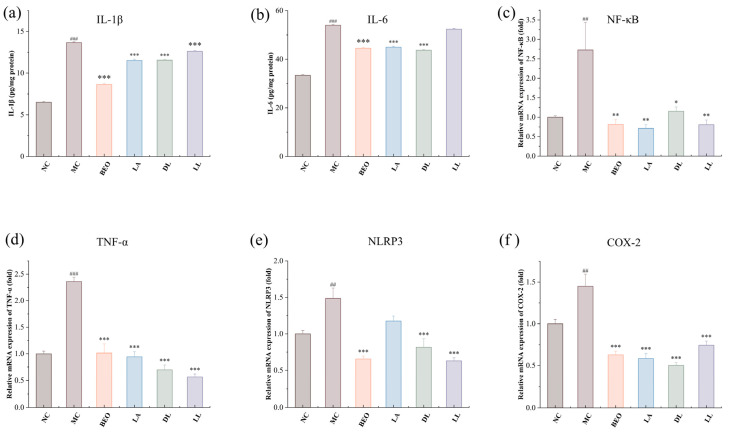
Effect of BEO and its main components on inflammation in muscle induced by progressive intense exercise. (**a**) IL-1β, (**b**) IL-6, (**c**) NF-κB, (**d**) TNF-α, (**e**) NLRP3, (**f**) COX-2. Values given are means ± SEMs, with *n* = 6. ^##^ *p* < 0.01 or ^###^ *p* < 0.001 indicates that the difference is significant compared with the NC group; * *p* < 0.05, ** *p* < 0.01 or *** *p* < 0.001 indicates that the difference is significant compared with the MC group.

Prolonged exercise and insufficient recovery are known to trigger systemic inflammation, particularly in skeletal muscle. To evaluate the inflammatory status, protein levels of the key pro-inflammatory cytokines IL-1β and IL-6 in skeletal muscle were measured. As shown in Figure 3a,b, both cytokines were significantly elevated in the MC group compared to the NC group (*p* < 0.001). Treatment with BEO and the three components significantly reduced IL-1β (*p* < 0.001), while IL-6 was significantly downregulated by BEO, LA, and DL (*p* < 0.001).

To further investigate the upstream molecular mechanism, the mRNA expression levels of several canonical inflammatory markers were examined, including NF-κB, TNF-α, NLRP3, and COX-2. Prolonged exercise significantly upregulated the expression of all four genes in the MC group (*p* < 0.01 for NF-κB, NLRP3, and COX-2; *p* < 0.001 for TNF-α; Figure 3c–f), confirming a robust inflammatory response at the transcriptional level. Long-term inhalation of BEO markedly suppressed the mRNA expression of all four genes (*p* < 0.01, *p* < 0.001, *p* < 0.001, *p* < 0.001, respectively). Among the individual components, LA significantly reduced the expression of NF-κB (*p* < 0.01), TNF-α (*p* < 0.001), and COX-2 (*p* < 0.001), but had a weaker effect on NLRP3. In contrast, both DL and LL significantly downregulated mRNA expression of NF-κB, TNF-α, NLRP3, and COX-2 (*p* < 0.05), with LL exhibiting effects comparable to those of BEO. These results indicate that BEO and its major components alleviate exercise-induced inflammation in skeletal muscle by reducing pro-inflammatory cytokine production at the protein level and the transcription of upstream inflammatory regulators. These effects suggest a strong anti-inflammatory role, likely through multi-target modulation.

### 2.4. Oxidative Stress Biomarkers in Skeletal Muscle

**Figure 4:**
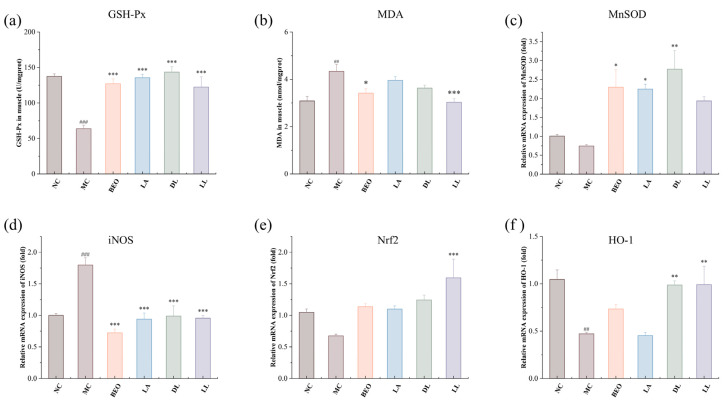
Effect of BEO and its main components on oxidative stress in muscle induced by progressive intense exercise. (**a**) GSH-Px, (**b**) MDA, (**c**) MnSOD, (**d**) iNOS, (**e**) Nrf2, (**f**) HO-1. Values given are means ± SEMs, with *n* = 6. ^##^ *p* < 0.01 or ^###^ *p* < 0.001 indicates that the difference is significant compared with the NC group; * *p* < 0.05, ** *p* < 0.01 or *** *p* < 0.001 indicates that the difference is significant compared with the MC group.

To evaluate antioxidant defense capacity, we examined markers of oxidative stress in skeletal muscle. Compared with the MC group, GSH-Px levels were significantly increased in all treatment groups (*p* < 0.001; Figure 4a), indicating enhanced enzymatic antioxidant activity. MDA, a marker of lipid peroxidation, was significantly reduced in the BEO (*p* < 0.05) and LL (*p* < 0.001) groups compared to the MC group (Figure 4b), reflecting decreased oxidative damage. At the transcriptional level, MnSOD expression was significantly upregulated in the BEO (*p* < 0.05), LA (*p* < 0.05), and DL (*p* < 0.01) groups (Figure 4c). In contrast, iNOS expression was significantly suppressed by all treatments (*p* < 0.001, Figure 4d), suggesting reduced nitric oxide-mediated stress. Notably, only the LL group exhibited a statistically significant increase in Nrf2 expression (*p* < 0.001, Figure 4e), while both DL and LL restored HO-1 expression (*p* < 0.01, Figure 4f), indicating potential activation of the Nrf2/HO-1 antioxidant pathway in these groups. These results suggest that BEO and its components mitigate oxidative stress via distinct complementary mechanisms.

### 2.5. Muscle Injury and Recovery-Related Biomarkers in Skeletal Muscle

**Figure 5:**
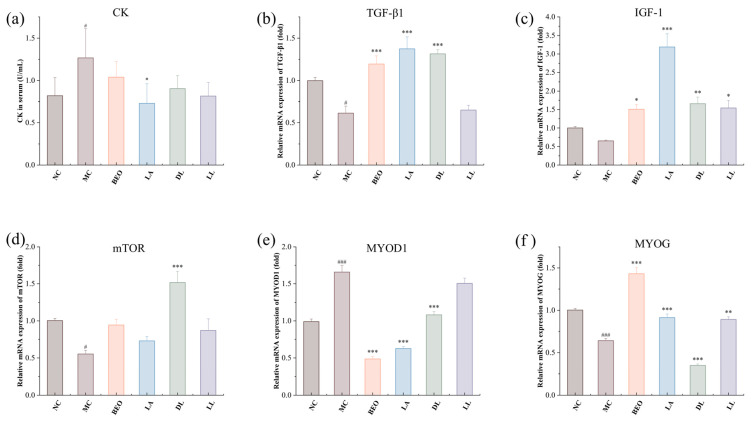
Effect of BEO and its main components on muscle injury and recovery. (**a**) CK, (**b**) TGF-β1, (**c**) IGF-1, (**d**) mTOR, (**e**) MYOD, (**f**) MYOG. Values given are means ± SEMs, with *n* = 6. ^#^ *p* < 0.05 or ^###^ *p* < 0.001 indicates that the difference is significant compared with the NC group; * *p* < 0.05, ** *p* < 0.01 or *** *p* < 0.001 indicates that the difference is significant compared with the MC group.

The protective effects of BEO and its main components against exercise-induced muscle damage, as well as their roles in promoting recovery, were further investigated. As shown in Figure 5a, the levels of CK, a marker of muscle membrane damage, were significantly elevated in the MC group (*p* < 0.05) and suppressed by LA treatment (*p* < 0.05). In contrast, the BEO, DL, and LL groups showed a downward trend that did not reach statistical significance, suggesting potential protective effects. TGF-β1 expression was significantly increased in the BEO, LA, and DL groups, whereas IGF-1 expression was significantly increased in all treatment groups compared with the MC group (*p* < 0.05; Figure 5b,c), indicating activation of muscle repair signaling. mTOR expression was significantly increased in the DL group (*p* < 0.001) and was moderately upregulated in the BEO, LA, and LL groups (*p* > 0.05; Figure 5d), which may influence downstream anabolic processes.

Notably, MYOD and myogenin (MYOG) are myogenic regulatory factors (MRFs), which modulate the processes of myogenesis and the repair of injured muscle [13]. Fatigue induced by exhaustive exercise led to a significant increase in MYOD1 (*p* < 0.001) and a decrease in MYOG mRNA expression (*p* < 0.001, Figure 5e,f), indicating differentiation despite satellite cell activation. Interestingly, BEO and LA treatment significantly reversed the expression pattern, significantly reducing MYOD1 levels while restoring MYOG expression (*p* < 0.001, Figure 5e,f). These findings suggest that BEO, particularly LA, may facilitate muscle recovery by alleviating damage and enhancing regenerative signaling and differentiation.

### 2.6. Western Blot Analysis of PI3K/AKT/mTOR/GSK3β Pathway in Skeletal Muscle

**Figure 6:**
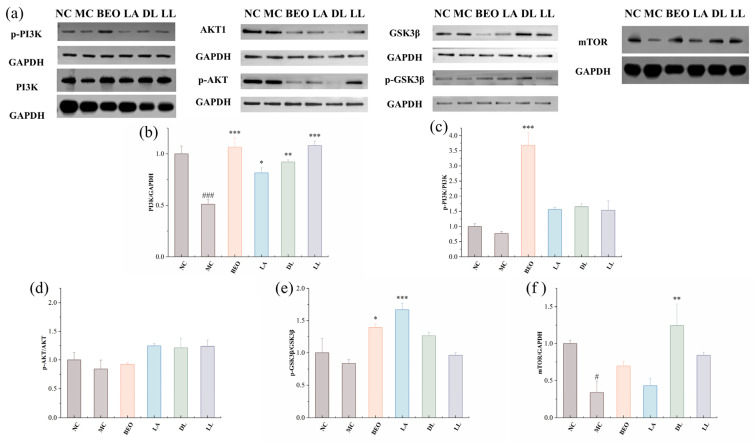
Effect of BEO and its main components on the PI3K/AKT/mTOR/GSK3β pathway. (**a**) Blot panel, (**b**) PI3K, (**c**) p-PI3K/PI3K ratio, (**d**) p-AKT (Ser473)/AKT, (**e**) p-GSK3β (Ser9)/GSK3β, (**f**) mTOR. Values given are means ± SEMs, with *n* = 3–4. ^#^ *p* < 0.05 or ^###^ *p* < 0.001 indicates that the difference is significant compared with the NC group; * *p* < 0.05, ** *p* < 0.01 or *** *p* < 0.001 indicates that the difference is significant compared with the MC group.

To further evaluate the molecular changes associated with fatigue recovery, we assessed selected proteins and phosphorylation ratios related to the PI3K/AKT/GSK3β/mTOR signaling pathway in skeletal muscle. As shown in Figure 6b, prolonged exercise significantly reduced total PI3K expression (*p* < 0.001), whereas all treatments significantly increased total PI3K levels (*p* < 0.001, *p* < 0.05, *p* < 0.01, *p* < 0.001, respectively). Phosphorylation analysis showed that only BEO significantly increased the p-PI3K/PI3K ratio compared with the MC group (*p* < 0.001, Figure 6c). AKT phosphorylation was evaluated by measuring the p-AKT (Ser473)/AKT ratio. Although the treatment groups showed an increasing tendency compared with the MC group, no significant differences were observed in the p-AKT/AKT ratio among groups (*p* > 0.05, Figure 6d), indicating that AKT phosphorylation was not robustly enhanced under the present conditions.

Since phosphorylation of GSK3β at Ser9 is associated with reduced GSK3β activity [14], p-GSK3β (Ser9) was examined. Compared with the MC group, BEO and LA significantly increased the p-GSK3β/GSK3β ratio (*p* < 0.05 and *p* < 0.001, respectively, Figure 6e), indicating enhanced inhibitory phosphorylation of GSK3β. In addition, total mTOR protein expression was significantly decreased in the MC group compared with the NC group (*p* < 0.05, Figure 6f). Among the treatment groups, only DL showed a significant increase compared with the MC group (*p* < 0.01). However, no significant differences were observed in the p-mTOR/mTOR ratio among groups (Supplementary Figure S2). Overall, these results indicate that BEO and its components differentially modulated selected PI3K/AKT/GSK3β/mTOR-related signaling markers during fatigue recovery.

## 3. Discussion

It is well-established that moderate exercise offers numerous health benefits. However, excessive exercise combined with inadequate recovery leads to systemic fatigue, characterized by inflammation, oxidative stress, and impaired muscle repair. In this study, we established a rat model of prolonged excessive exercise-induced fatigue through an 8-week protocol consisting of progressive treadmill exercise followed by forced swimming. We demonstrated that long-term inhalation of BEO and its main constituents (linalyl acetate, D-limonene, and linalool) improved exercise performance and ameliorated fatigue-related biochemical alterations. These effects were achieved through coordinated regulation of peripheral mechanisms, including oxidative defense, inflammation suppression, muscle regeneration and metabolic signaling. In addition to the fatigue-related protective effects, BEO significantly increased the liver index compared with both the NC and MC groups. This finding suggests that prolonged exposure to the complete BEO mixture may induce a hepatic response. One possible explanation is that repeated inhalation of the complex volatile mixture, including minor constituents, increased hepatic biotransformation and metabolic demands, potentially leading to adaptive hepatic enlargement [15].

Prolonged exercise-induced physical fatigue is closely accompanied by oxidative stress, arising from excessive generation of reactive oxygen and nitrogen species (ROS/RNS) during muscular exertion and tissue damage [9,16]. In this study, BEO and its main components significantly enhanced antioxidant defenses, as reflected by the increased GSH-Px activity and reduced MDA accumulation in skeletal muscle (Figure 4a,b). Moreover, MnSOD expression was upregulated by BEO, LA, and DL (Figure 4c), suggesting the restoration of mitochondrial redox homeostasis. This finding aligns with previous reports showing that inhaled essential oil mixtures improved exhaustion time and oxidative stress-related fatigue markers [17]. In contrast, iNOS, a major contributor to RNS production under inflammation and stress conditions [18], was significantly suppressed by BEO and all three components (Figure 4d). Notably, LL treatment selectively upregulated the Nrf2/HO-1 signaling pathway, whereas LA and DL primarily influenced antioxidant enzyme expression and iNOS suppression. These differences suggest differential engagement of oxidative stress response mechanisms among the individual components.

Inflammation is a critical pathological feature of excessive exercise-induced fatigue. Persistent inflammatory responses, if left uncontrolled, can lead to tissue damage and functional decline in skeletal muscle [19]. Histological analysis revealed substantial inflammatory infiltration in the skeletal muscle of fatigued rats, which was markedly attenuated by intervention with BEO and its components. To evaluate the inflammatory response at the protein level, we first measured the concentrations of two key pro-inflammatory cytokines, IL-1β and IL-6, in muscle. IL-1β was significantly reduced by BEO, LA, DL, and LL, whereas IL-6 was significantly reduced by BEO, LA, and DL, supporting the anti-inflammatory effects of these interventions. To further explore the underlying mechanisms, we assessed the mRNA expression of several upstream regulators and signaling molecules, including NF-κB, TNF-α, COX-2, and NLRP3. All four genes were significantly downregulated in the BEO, DL, and LL groups, whereas LA exhibited relatively limited impact on NLRP3, suggesting selective modulation of inflammasome-related pathways. The evidence demonstrates that BEO alleviates prolonged exercise-induced inflammation through multi-level modulation, thereby highlighting its capacity to mitigate fatigue-related tissue stress. This is also consistent with studies on other plant-derived bioactives, such as Lonicera caerulea berry polyphenols, which alleviate exercise fatigue by suppressing both oxidative stress and inflammation in skeletal muscle [20].

Exercise-induced muscle damage triggers a complex regenerative process involving satellite cell activation, inflammatory cytokine signaling, and protein synthesis [21]. Previous studies have generally reported parallel upregulation of MYOD and MYOG as hallmarks of activated myogenesis [22,23]. Interestingly, our results reveal that BEO and LA reversed the abnormal MYOD1/MYOG expression pattern by reducing MYOD1 expression while restoring MYOG expression. This pattern suggests that BEO may prevent aberrant or prolonged satellite cell activation while promoting orderly differentiation [13,24]. Additionally, the observed upregulation of TGF-β1, IGF-1, and mTOR at the transcriptional level further supports active anabolic engagement. While the IGF-1 pathway is widely studied in nutritional interventions, the observed regulation of IGF-1/mTOR-related markers is consistent with the established roles of IGF-1/PI3K/AKT/mTOR and IGF-1/PI3K/AKT/GSK3β signaling in skeletal muscle anabolic regulation [25].

Accumulating evidence indicates that the PI3K/AKT-related signaling pathway is essential for maintaining metabolic homeostasis and alleviating exercise-induced fatigue [9]. Skeletal muscle is a primary site of glucose uptake and glycogen storage during physical activity [26], while downstream effectors such as GSK3β and mTOR are involved in glycogen, protein synthesis, and muscle adaptation [9]. In the present study, all four treatments significantly increased total PI3K protein expression, whereas only BEO significantly increased the p-PI3K/PI3K ratio. This finding suggests that BEO exerted a more pronounced effect on PI3K phosphorylation than its individual components. However, the p-AKT/AKT ratio was not significantly altered among groups. AKT phosphorylation in skeletal muscle is known to be rapid and transient after exercise, with its magnitude varying considerably depending on the exercise protocol and the timing of tissue collection [27,28]. Therefore, the absence of a significant change in p-AKT/AKT at the selected sampling time point may partly reflect that a transient phosphorylation response was not captured. Notably, BEO and LA specifically increased GSK3β phosphorylation at Ser9, suggesting enhanced inhibition of GSK3β activity. Previous evidence suggests that contraction-associated GSK3β phosphorylation may not always occur in parallel with AKT phosphorylation [29]. Thus, the increase in GSK3β inhibitory phosphorylation despite the unchanged p-AKT/AKT ratio may reflect differential temporal regulation or the involvement of additional regulatory mechanisms. In addition, DL increased total mTOR protein expression, whereas the p-mTOR/mTOR ratio remained unchanged, suggesting an effect on mTOR protein abundance rather than phosphorylation-dependent activation. Collectively, these findings indicate that BEO and its major components differentially regulated selected PI3K/AKT/GSK3β/mTOR-related markers, with BEO showing prominent effects on PI3K and GSK3β phosphorylation.

Natural active ingredients have been reported to alleviate exercise fatigue through coordinated regulation of oxidative stress, inflammation, energy metabolism, and recovery-associated signaling networks [9]. Our findings were in line with this current view. Compared with the individual monomers, BEO exerted broader effects across endurance, inflammation, oxidative stress, and muscle recovery-related markers. This pattern suggests that BEO activity may not be fully explained by a single major constituent but rather reflects the combined contributions of linalyl acetate, D-limonene, linalool, and other minor volatile components.

Several limitations should be acknowledged. Firstly, although BEO increased the p-PI3K/PI3K ratio and enhanced the inhibitory phosphorylation of GSK3β, no significant changes were observed in the p-AKT/AKT or p-mTOR/mTOR ratios. Given the dynamic and time-dependent nature of protein phosphorylation, analyses at multiple sampling time points are needed to more comprehensively characterize the temporal regulation of this signaling network. In addition, pathway-specific intervention studies are required to further establish the causal involvement of PI3K/GSK3β-related signaling in the effects of BEO. Secondly, further studies should include liver function biomarkers, histological analysis, and metabolic profiling to clarify the hepatic effects and safety implications of prolonged BEO exposure. Thirdly, dose–response evaluation should be included in future studies to clarify the optimal exposure dose and safety range of prolonged BEO inhalation. Fourth, several inflammation-, oxidative-stress-, and myogenesis-related markers were assessed, but primarily at the transcriptional level; therefore, broader protein-level validation is needed in future studies to further confirm these regulatory mechanisms. Fifth, it should be noted that the present study primarily focused on long-term and post-exercise inhalation. Further studies are needed to examine the time-dependent efficacy of BEO across different fatigue and recovery stages, and to elucidate how olfactory perception is translated into systemic metabolic responses.

## 4. Materials and Methods

### 4.1. Materials and Reagents

Bergamot essential oil was purchased from Ecoarts Enterprise Co., Ltd. (Shanghai, China); its detailed GC-MS profile is provided in Supplementary S1. D-limonene (≥95%), linalyl acetate (≥96%), and linalool (≥98%) were purchased from Macklin Inc. (Wuhan, China) and Aladdin Scientific (Shanghai, China). Assay kits for glutathione peroxidase (GSH-Px), malondialdehyde (MDA), and creatine kinase (CK) were obtained from Nanjing Jiancheng Bioengineering Institute (Nanjing, China). ELISA kits for IL-1β and IL-6 were supplied by Shanghai Hengyuan Biological Technology Co., Ltd. (Shanghai, China). RT-qPCR reagents were acquired from Vazyme International LLC (Nanjing, China). All primary and secondary antibodies for Western blotting (detailed in Section 4.7) were procured from ABclonal Inc. (Wuhan, China), Sino Biological Inc. (Beijing, China), Proteintech Group Inc. (Wuhan, China), and Affinity Biosciences LTD (Cincinnati, OH, USA). All other chemicals were of analytical grade.

### 4.2. Animal Experiments

Sixty specific-pathogen-free (SPF) male Sprague Dawley rats (150 ± 10 g) were obtained from Hunan SJA Laboratory Animals Co., Ltd. (Hunan, China, Approval No. SYXK (E) 2020-0084). Rats were housed under standard laboratory conditions (24 ± 2 °C, 40–70% relative humidity, 12 h light/dark cycle) with ad libitum access to standard chow and distilled water. All procedures strictly complied with the Guidelines for the Care and Use of Laboratory Animals and were approved by the Animal Ethics Committee of Huazhong Agricultural University (HZAAURA-2024-0034; approved on 1 September 2024).

**Figure 7:**
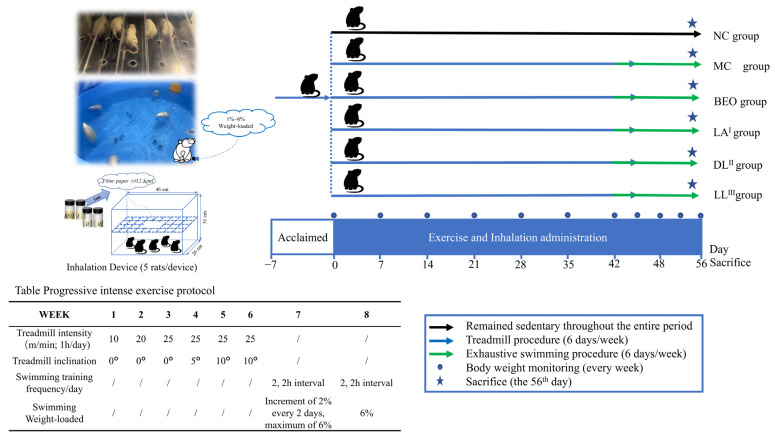
Experimental protocol for the effects of inhalation of the BEO and its main components on progressive intense exercise-induced fatigue. I, II, and III indicate linalyl acetate, D-limonene, and linalool, respectively.

Following a one-week acclimation, the rats were randomly assigned to six groups (Figure 7): Normal Control (NC), Model Control (MC), BEO, linalyl acetate (LA), D-limonene (DL), and linalool (LL). To establish the chronic fatigue model, all groups except NC underwent a progressive high-intensity exercise protocol consisting of 6 weeks of treadmill running followed by 2 weeks of swimming training. Non-compliant rats were excluded while ensuring at least six biological replicates per group for final analyses.

For the inhalation intervention, rats in the treatment groups (BEO, LA, DL, LL) were placed in semi-enclosed devices (46 cm × 31 cm × 26 cm, length × height × width) after each daily exercise session. Filter papers containing 1 mL of the respective volatile agents were placed on a central partition to ensure even volatilization at room temperature (24 ± 2 °C). The exposure lasted for 2 h and was performed daily, following previous protocols [7].

### 4.3. Exhaustive Swimming Test

Swimming training was conducted in a Morris water maze pool for 30 min, twice daily (with a 2 h interval). To induce progressive fatigue, a weight corresponding to 2% of the rat’s body weight was attached to its tail base, with the weight increased by 2% every 2 days until reaching a maximum of 6%. Water temperature was maintained at 25 ± 2 °C with constant stirring. At the end of the 7th week, a 6% weight-loaded swimming exhaustion test was performed. Exhaustion was defined as the inability to keep the head above water for 10 s, and the time to exhaustion was recorded to evaluate endurance performance.

### 4.4. Sample Collection and Preparation

Following the final swimming session, rats were fasted for 12 h and anesthetized with ether. Blood was collected and centrifuged (1123× *g*, 10 min, 4 °C) to separate serum. The gastrocnemius muscle and major organs (liver, spleen, lung) were rapidly excised, weighed to calculate organ coefficients (organ weight/body weight × 100), flash-frozen in liquid nitrogen, and stored at −80 °C.

### 4.5. Hematoxylin and Eosin (H&E) Staining and Quantification

Gastrocnemius muscle tissues were fixed in 4% paraformaldehyde overnight, dehydrated, embedded in paraffin, and sectioned (3–5 μm). Sections were deparaffinized in xylene, rehydrated through a graded ethanol series, and stained with Hematoxylin and Eosin (H&E) for morphological evaluation. Quantitative histological analysis of H&E-stained skeletal muscle sections was performed using Fiji (ImageJ, version 1.54p; National Institutes of Health, Bethesda, MD, USA). Non-overlapping fields were selected from structurally intact regions while avoiding folds, torn areas, large vessels, staining artifacts, and poorly preserved regions. The inflammatory infiltration area was manually outlined as clusters of small round hematoxylin-positive cells in the interstitial regions and expressed as a percentage of the total field area. Muscle fiber CSA was measured by outlining clearly identifiable transversely sectioned fibers with intact boundaries, while obliquely sectioned, incomplete, damaged, or poorly defined fibers were excluded. Data from multiple fields or fibers within the same sample were averaged for each biological replicate before statistical analysis.

### 4.6. RT-qPCR Analysis

Total RNA was extracted from the gastrocnemius muscle using a commercial isolation kit, and its concentration and purity (A260/280 ratio) were verified by spectrophotometry. RNA was reverse-transcribed into cDNA using the All-in-One First-Strand cDNA Synthesis SuperMix. Quantitative real-time PCR was performed using a CFX96 system (Bio-Rad, Hercules, CA, USA) utilizing the 2× ChamQ Universal SYBR qPCR Master Mix in a 20 μL reaction volume. Relative gene expression was calculated using the 2^−ΔΔCt^ method, with GAPDH serving as the endogenous control. Primer sequences are listed in Supplementary S3.

### 4.7. Western Blot Analysis

Gastrocnemius muscle tissues were homogenized in ice-cold lysis buffer, incubated on ice for 30 min, and centrifuged (14,000× *g*, 15 min, 4 °C). Supernatants were collected, and protein concentrations were quantified using a BCA protein assay kit (Haimen, Jiangsu, China). Equal amounts of protein (40 μg) were resolved by SDS-PAGE and transferred onto PVDF membranes (Merck Millipore, Cork, Ireland). Membranes were blocked (20 mM Tris-base, 137 mM NaCl, 1% BSA, 1% Tween-20, 0.1% sodium azide) and incubated overnight at 4 °C with primary antibodies targeting PI3K (1:10,000), p-PI3K (1:1000), p-AKT (1:1000), AKT1 (1:1000), p-GSK3β (1:1000), GSK3β (1:5000), mTOR (1:1600), p-mTOR (1:2000), and GAPDH (1:100,000). After TBST washes, membranes were incubated with appropriate HRP-conjugated secondary antibodies. Protein bands were visualized using enhanced chemiluminescence (Abkine, Wuhan, China) and quantified via ImageJ 1.54J.

### 4.8. Statistical Analysis

Data are presented as the mean ± standard error (S.E.). Statistical significance was determined by One-Way Analysis of Variance (ANOVA) followed by Tukey’s Honestly Significant Difference (HSD) post hoc test using SPSS 26.0 (SPSS Inc., Chicago, IL, USA). A value of *p* < 0.05 was considered statistically significant. In figures, ^#^ *p* < 0.05, ^##^ *p* < 0.01, or ^###^ *p* < 0.001 indicate that the difference is significant compared with the NC group; * *p* < 0.05, ** *p* < 0.01, or *** *p* < 0.001 indicate that the difference is significant compared with the MC group. All graphs were generated using OriginPro 2024 (Northampton, MA, USA) and Microsoft PowerPoint (Redmond, WA, USA).

## 5. Conclusions

Taken together, our investigation revealed that BEO effectively ameliorated inflammation, oxidative stress, and muscle injury associated with prolonged exercise-induced fatigue. These protective effects were accompanied by recovery-associated molecular changes, including increased Igf1 expression, restored total PI3K protein expression, enhanced PI3K phosphorylation, and increased inhibitory phosphorylation of GSK3β, suggesting that modulation of PI3K/GSK3β-related signaling may contribute to improved skeletal muscle recovery. The broader regulatory effect of BEO may be related to its multi-component composition, which may provide complementary contributions beyond those of individual major constituents. Overall, these results support the potential of plant food-derived BEO as a multifunctional, aroma-based strategy for managing physical fatigue, with its chemical synergy representing a key determinant of its health benefits.

## Figures and Tables

**Table 1:** Effect of BEO and the main components on organ indices in rats. Values are means ± SEMs, with *n* = 6. ^#^ *p* < 0.05 indicates a significant difference compared with the NC group; * *p* < 0.05 indicates a significant difference compared with the MC group.

Group	Liver Index (%)	Spleen Index (%)	Lung Index (%)
NC	2.44 ± 0.14	0.17 ± 0.01	0.44 ± 0.03
MC	2.46 ± 0.06	0.16 ± 0.01	0.48 ± 0.01
BEO	2.63 ± 0.07 ^#^*	0.15 ± 0.01	0.47 ± 0.03
LA	2.49 ± 0.05	0.15 ± 0.01	0.45 ± 0.03
DL	2.54 ± 0.10	0.17 ± 0.01	0.49 ± 0.03
LL	2.49 ± 0.13	0.16 ± 0.01	0.46 ± 0.05

## Data Availability

Data will be available on reasonable request.

## Supplementary Materials

The following supporting information can be downloaded at: https://www.mdpi.com/article/10.3390/ijms27156736/s1.

## Abbreviations

The following abbreviations are used in this manuscript:

AKT	Protein Kinase B
BCAAs	branched-chain amino acids
BEO	Bergamot essential oil
CEOs	Citrus essential oils
CK	Creatine kinase
COX-2	Cyclooxygenase-2
DL	D-limonene
EOs	Essential oils
GRAS	Generally recognized as safe
GSH-Px	Glutathione peroxidase
GSK3β	Glycogen synthase kinase-3β
H&E	Hematoxylin and eosin
HO-1	Heme oxygenase-1
IGF-1	Insulin-like growth factor 1
iNOS	Inducible nitric oxide synthase
LA	Linalyl acetate
LL	Linalool
MC	Model control
MDA	Malondialdehyde
MnSOD	Manganese superoxide dismutase
mTOR	Mammalian target of rapamycin
MYOD	Myogenic differentiation 1
MYOG	Myogenin
NC	Normal control
NF-κB	Nuclear factor kappa B
NLRP3	NOD-, LRR- and pyrin domain-containing protein 3
Nrf2	Nuclear factor erythroid 2-related factor 2
p-AKT	Phosphorylation of Akt
p-GSK3β	Phosphorylated GSK3β
PI3K	Phosphoinositide 3-kinase
ROS/RNS	Reactive oxygen and nitrogen species
TBST	Tris-buffered saline with tween-20
TGF-β	Transforming growth factor beta
TNF-α	Tumor necrosis factor-α

## References

[1] Liu X., Liu J. (2026). Research progress on exercise fatigue from the perspective of fatigue biomarkers. PeerJ.

[2] Mukund K., Subramaniam S. (2020). Skeletal muscle: A review of molecular structure and function, in health and disease. Wiley Interdiscip. Rev. Syst. Biol. Med..

[3] Ma X., Chen H., Cao L., Zhao S., Zhao C., Yin S., Hu H. (2021). Mechanisms of Physical Fatigue and its Applications in Nutritional Interventions. J. Agric. Food Chem..

[4] Jiang X., Muthusamy K., Fang X. (2024). A scoping review of olfactory interventions for fatigue relief: Addressing occupational health hazards. Front. Public Health.

[5] Ruan Q., Dai C., Lin Y., Wu W., Yi F. (2026). Aroma Olfactory Intervention: Enhancing Stress Recovery via Brain Neural Activity Modulation. Buildings.

[6] Zhang W., Shi R., Gao T., Hu Y., Zhou J., Li C., Wang P., Yang H., Xing W., Dong L. (2023). Repeated Inhalation of Peppermint Essential Oil Improves Exercise Performance in Endurance-Trained Rats. Nutrients.

[7] Tian L., Hu T., Zhang S., Zhang H., Yang C., Chen G., Pan S. (2022). A Comparative Study on Relieving Exercise-Induced Fatigue by Inhalation of Different Citrus Essential Oils. Molecules.

[8] Meng Q., Su C.H. (2025). Antioxidant Defense and Redox Signaling in Elite Soccer Players: Insights into Muscle Function, Recovery, and Training Adaptations. Antioxidants.

[9] Zhao R., Wu R., Jin J., Ning K., Wang Z., Yi X., Kapilevich L., Liu J. (2023). Signaling pathways regulated by natural active ingredients in the fight against exercise fatigue—A review. Front. Pharmacol..

[10] Dosoky N.S., Setzer W.N. (2018). Biological Activities and Safety of *Citrus* spp. Essential Oils. Int. J. Mol. Sci..

[11] Fung T.K.H., Lau B.W.M., Ngai S.P.C., Tsang H.W.H. (2021). Therapeutic Effect and Mechanisms of Essential Oils in Mood Disorders: Interaction between the Nervous and Respiratory Systems. Int. J. Mol. Sci..

[12] Koyama S., Heinbockel T. (2020). The Effects of Essential Oils and Terpenes in Relation to Their Routes of Intake and Application. Int. J. Mol. Sci..

[13] Fernández-Lázaro D., Garrosa E., Seco-Calvo J., Garrosa M. (2022). Potential Satellite Cell-Linked Biomarkers in Aging Skeletal Muscle Tissue: Proteomics and Proteogenomics to Monitor Sarcopenia. Proteomes.

[14] Wang L., Li J., Di L.J. (2022). Glycogen synthesis and beyond, a comprehensive review of GSK3 as a key regulator of metabolic pathways and a therapeutic target for treating metabolic diseases. Med. Res. Rev..

[15] Satou T., Takahashi M., Kasuya H., Murakami S., Hayashi S., Sadamoto K., Koike K. (2013). Organ accumulation in mice after inhalation of single or mixed essential oil compounds. Phytother. Res..

[16] Powers S.K., Nelson W.B., Hudson M.B. (2011). Exercise-induced oxidative stress in humans: Cause and consequences. Free Radic. Biol. Med..

[17] Li Z., Wu F., Shao H., Zhang Y., Fan A., Li F. (2017). Does the Fragrance of Essential Oils Alleviate the Fatigue Induced by Exercise? A Biochemical Indicator Test in Rats. Evid. Based Complement. Altern. Med..

[18] Wink D.A., Hines H.B., Cheng R.Y., Switzer C.H., Flores-Santana W., Vitek M.P., Ridnour L.A., Colton C.A. (2011). Nitric oxide and redox mechanisms in the immune response. J. Leukoc. Biol..

[19] Bouredji Z., Argaw A., Frenette J. (2022). The inflammatory response, a mixed blessing for muscle homeostasis and plasticity. Front. Physiol..

[20] Liu S., Meng F., Zhang D., Shi D., Zhou J., Guo S., Chang X. (2022). Lonicera caerulea Berry Polyphenols Extract Alleviates Exercise Fatigue in Mice by Reducing Oxidative Stress, Inflammation, Skeletal Muscle Cell Apoptosis, and by Increasing Cell Proliferation. Front. Nutr..

[21] Stožer A., Vodopivc P., Križančić Bombek L. (2020). Pathophysiology of exercise-induced muscle damage and its structural, functional, metabolic, and clinical consequences. Physiol. Res..

[22] Yu D., Cai Z., Li D., Zhang Y., He M., Yang Y., Liu D., Xie W., Li Y., Xiao W. (2021). Myogenic Differentiation of Stem Cells for Skeletal Muscle Regeneration. Stem Cells Int..

[23] Tsuji R., Hayashi T., Takahashi S., Asakura A., Fujita R. (2025). The multifaceted role of MyoD in adult skeletal muscle: Homeostasis, regeneration, and diseases. Am. J. Physiol. Cell Physiol..

[24] Blum J., Epstein R., Watts S., Thalacker-Mercer A. (2021). Importance of Nutrient Availability and Metabolism for Skeletal Muscle Regeneration. Front. Physiol..

[25] Rommel C., Bodine S.C., Clarke B.A., Rossman R., Nunez L., Stitt T.N., Yancopoulos G.D., Glass D.J. (2001). Mediation of IGF-1-induced skeletal myotube hypertrophy by PI(3)K/Akt/mTOR and PI(3)K/Akt/GSK3 pathways. Nat. Cell Biol..

[26] Merz K.E., Thurmond D.C. (2020). Role of Skeletal Muscle in Insulin Resistance and Glucose Uptake. Compr. Physiol..

[27] Camera D.M., Edge J., Short M.J., Hawley J.A., Coffey V.G. (2010). Early time course of Akt phosphorylation after endurance and resistance exercise. Med. Sci. Sports Exerc..

[28] Dreyer H.C., Fujita S., Glynn E.L., Drummond M.J., Volpi E., Rasmussen B.B. (2010). Resistance exercise increases leg muscle protein synthesis and mTOR signalling independent of sex. Acta Physiol..

[29] Sakamoto K., Hirshman M.F., Aschenbach W.G., Goodyear L.J. (2002). Contraction Regulation of Akt in Rat Skeletal Muscle. J. Biol. Chem..

