# Supplementary material for: Long-Term Bergamot Essential Oil Inhalation Alleviates Prolonged Exercise-Induced Fatigue in Rats Through the Modulation of Skeletal Muscle Inflammation, Oxidative Stress, and Regeneration-Related Signaling
Source: Int J Mol Sci. 2026 Jul 28;27(15):6736. doi: 10.3390/ijms27156736 (PMC13467510; doi:10.3390/ijms27156736)
Supplement: Supplementary file 1 [file ijms-27-06736-s001.zip › ijms-4405352-supplementary.pdf]

Supplementary Material

Long-Term Bergamot Essential Oil Inhalation Alleviates Prolonged Exercise-Induced Fatigue in Rats Through the Modulation of Skeletal Muscle Inflammation, Oxidative Stress, and Regeneration-Related Signaling

Lei Tian[1,2,3,4]; Kaixin Shi[5]; Siyi Pan[1,2,3*].

[1] College of Food Science and Technology, Huazhong Agricultural University, Wuhan, Hubei 430070, China;

[2] Key Laboratory of Environment Correlative Dietology, Ministry of Education, Huazhong Agricultural University, Wuhan, Hubei 430070, China;

[3] Key Laboratory of Fruit & Vegetable Processing & Quality Control, Huazhong Agricultural University, Wuhan, Hubei 430070, China;

[4] Department of Cell Biology and Human Anatomy, University of California at Davis, School of Medicine, Davis, CA, 95616, USA

[5] Agricultural Products Processing Research Institute, Chongqing Academy of Agricultural Science, Chongqing 401329, China

S1 GC-MS Analysis of Bergamot Essential Oil

Figure S1 Chromatogram of Bergamot essential oil

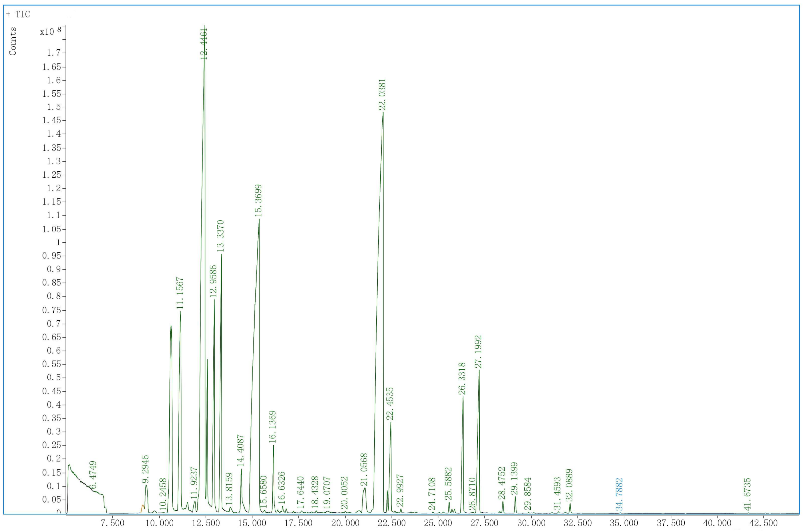

Table S1 Detected compounds of BEO （GC-MS data were analyzed using Agilent MassHunter Unknowns Analysis software, with compound identification performed based on the NIST20 library. Only compounds with a match score greater than 80 were selected）

RT	Compound	Area%	Molecular Fomula
22.04	Linalyl acetate	26.41	$C_{12}H_{20}O_2$
12.45	D-Limonene	19.92	$C_{10}H_{16}$
15.37	Linalool	19.42	$C_{10}H_{18}O$
10.63	Cyclohexane, 1-methylene-4-(1-methylethenyl)-	5.44	$C_{10}H_{16}$
26.33	2,6-Octadien-1-ol, 3,7-dimethyl-, acetate, (Z)-	5.05	$C_{12}H_{20}O_2$

11.16	Bicyclo[3.1.1] heptane, 6,6-dimethyl-2-methylene-, (1S)-	4.65	$C_{10}H_{16}$
13.34	gamma-Terpinene	4.19	$C_{10}H_{16}$
12.96	3-Carene	3.48	$C_{10}H_{16}$
12.58	(1R)-2,6,6-Trimethylbicyclo [3.1.1] hept-2-ene	2.71	$C_{10}H_{16}$
22.45	Citral	1.60	$C_{10}H_{16}O$
21.06	Neral	0.93	$C_{10}H_{16}O$
16.14	2,4,6-Octatriene, 2,6-dimethyl-, (E, Z)-	0.86	$C_{10}H_{16}$
14.41	1,3-Cyclohexadiene, 1-methyl-4-(1-methylethyl)-	0.82	$C_{10}H_{16}$
22.26	.alpha.-Methyl-.alpha.-[4-methyl-3-pentenyl]oxiranemethanol	0.22	$C_{10}H_{18}O_2$
9.12	Bicyclo[3.1.0]hex-2-ene, 2-methyl-5-(1-methylethyl)-	0.21	$C_{10}H_{16}$
11.92	(+)-4-Carene	0.21	$C_{10}H_{16}$
29.14	cis-.alpha.-Bergamotene	0.21	$C_{15}H_{24}$
11.53	.alpha.-Phellandrene	0.17	$C_{10}H_{16}$
28.48	Bicyclo[5.2.0]nonane, 2-methylene-4,8,8-trimethyl-4-vinyl-	0.15	$C_{15}H_{24}$

13.82	Ethyl 2-(5-methyl-5-vinyltetrahydrofuran-2-yl)propan-2-yl carbonate	0.14	$C_{13}H_{22}O_4$
25.59	.alpha.-Terpinyl acetate	0.13	$C_{12}H_{20}O_2$
32.09	1H-Benzocycloheptene, 2,4a,5,6,7,8,9,9a-octahydro-3,5,5-trimethyl-9-methylene-, (4aS-cis)-	0.12	$C_{15}H_{24}$
16.63	1,3-Cyclopentadiene, 1,2,3,4,5-pentamethyl-	0.10	$C_{10}H_{16}$
20.74	Cyclohexanol, 1-methyl-4-(1-methylethenyl)-	0.08	$C_{10}H_{18}O$
9.74	Camphene	0.06	$C_{10}H_{16}$
25.87	3-Nonanol, 1,2:6,7-diepoxy-3,7-dimethyl-, acetate	0.06	$C_{13}H_{22}O_4$
25.74	3-Nonanol, 1,2:6,7-diepoxy-3,7-dimethyl-, acetate	0.06	$C_{13}H_{22}O_4$
16.82	(+)-2-Bornanone	0.05	$C_{10}H_{16}O$
16.37	cis-(-)-1,2-Epoxy-p-menth-8-ene	0.05	$C_{10}H_{16}O$
22.99	p-Menth-8-en-3-ol, acetate	0.05	$C_{12}H_{20}O_2$
17.64	cis-Chrysanthenol	0.04	$C_{10}H_{16}O$
20.01	Fenchyl acetate	0.04	$C_{12}H_{20}O_2$

15.66	2,6,6-Trimethylcyclohexa-1,4-dienecarbaldehyde	0.03	$C_{10}H_{14}O$
18.43	3,6-Octadienal, 3,7-dimethyl-	0.03	$C_{10}H_{16}O$
28.31	cis-.alpha.-Bergamotene	0.02	$C_{15}H_{24}$
22.67	Cyclohexanol, 1-methyl-4-(1-methylethylidene)-	0.02	$C_{10}H_{18}O$
16.54	(+)-(E)-Limonene oxide	0.02	$C_{10}H_{16}O$
17.92	2H-Pyran-3-ol, 6-ethenyltetrahydro-2,2,6-trimethyl-	0.02	$C_{10}H_{18}O_2$
31.46	Naphthalene, 1,2,3,5,6,7,8,8a-octahydro-1,8a-dimethyl-7-(1-methylethenyl)-, [1R-(1.alpha.,7.beta.,8a.alpha.)]-	0.02	$C_{15}H_{24}$
18.83	L-.alpha.-Terpineol	0.01	$C_{10}H_{18}O$
23.54	2-Cyclohexen-1-ol, 3-methyl-6-(1-methylethyl)-, acetate	0.01	$C_{12}H_{20}O_2$
26.87	Cyclohexanol, 2-methyl-5-(1-methylethenyl)-	0.01	$C_{10}H_{18}O$
29.86	1,4,7,-Cycloundecatriene, 1,5,9,9-tetramethyl-, Z,Z,Z-	0.01	$C_{15}H_{24}$

31.12	Bicyclo[7.2.0]undec-4-ene, 4,11,11-trimethyl-8-methylene-	0.01	$C_{15}H_{24}$
20.22	2-Cyclohexen-1-ol, 2-methyl-5-(1-methylethenyl)-, cis-	0.01	$C_{10}H_{16}O$
15.89	2-Cyclohexen-1-ol, 1-methyl-4-(1-methylethenyl)-, trans-	0.01	$C_{10}H_{16}O$
34.79	1H-Cycloprop[e]azulen-7-ol, decahydro-1,1,7-trimethyl-4-methylene-, [1ar-(1a.alpha.,4a.alpha.,7.beta.,7a.beta.,7b.alpha.)]-	0.01	$C_{15}H_{24}O$

S2 p-mTOR/mTOR ratio

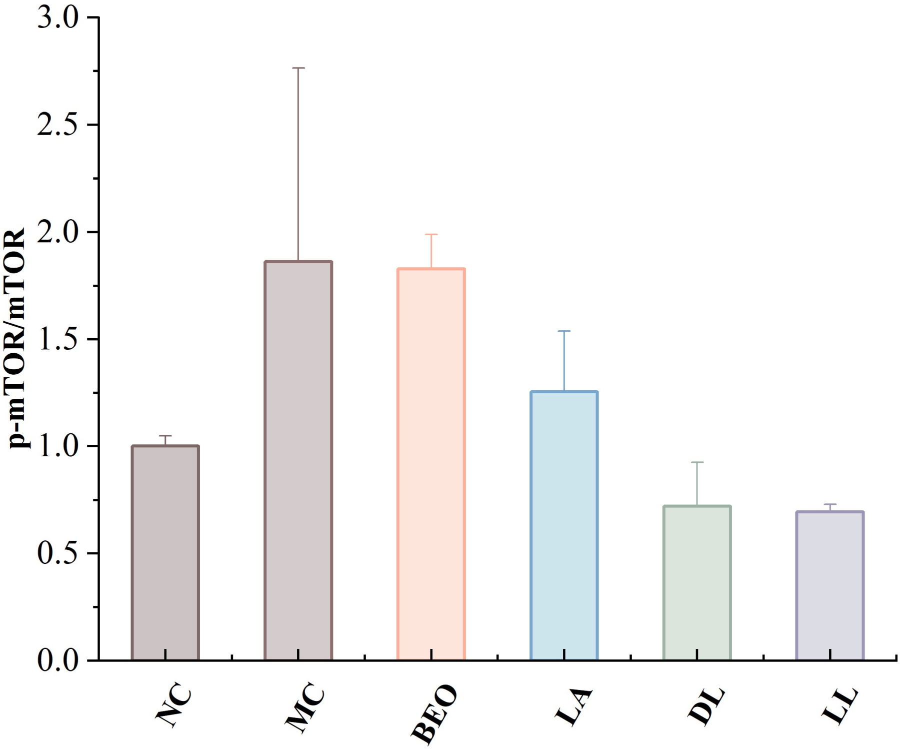

Figure S2 Effect of BEO and its main components on p-mTOR/mTOR ratio. Values given are means ± SEM, with n = 3-4.

S3 Primer Sequence for RT-qPCR

Table S2 Primer Sequence for RT-qPCR

	Forward primer (5′ → 3′)	Reverse primer (5′ → 3′)
Gapdh/GAPDH	ATGACTCTACCCACGGCAAG	CTGGAAGATGGTGATGGGTT
Tnf/TNF-α	TTCTCATTCCTGCTCGTGG	GCTTGGTGGTTTGCTACG
Nfkb1/NF-κB	GTCACCAAAGACCCACCTCAC	AGGTCTCGCTTCTTCACACAC
Nlrp3/NLRP3	AGACCTCCAAGACCACGACTG	TTCCATCCGCAGCCAATGAAC

Il6/IL-6	TCACAGAAGGAGTGGCTAAG	TCTGACCACAGTGAGGAATG
Il1b/IL-1β	GACAGACCCCAAAAGATTAAG	TCCACGGGCAAGACATAG
Nos2/iNOS	TGATCTTGTGCTGGAGGTGACCAT	TGTAGCGCTGTGTGTCACAGAAGT
Ptgs2/COX-2	AATCGCTGTACAAGCAGTGG	GCAGCCATTTCTTTCTCTCC
Sod2/MnSOD	CGTGCTCCCACACATCAATC	TGAACGTCACCGAGGAGAAG
Nfe2l2/Nrf2	TGGACTTGGAATTGCCACCG	TCCAGCTCATAATCCTTCTGTCG
Hmox1/HO-1	CCACGCATATACCCGCTACC	ACTTGGTGGGGTTGTCGATG
Myod1/MYOD1	CTGCCTGTCCAGCATAGTG	CATCGCTTGAGGATGTCTCC
Myog/MYOG	TGCCATCCAGTACATTGAGC	CTGTGGGAGTTGCATTCACT
Igf1/IGF-1	ACATCTCTTCTACCTGGCACTCTG	CCTTGGTCCACACACGAACTG
Tgfb1/TGF-β	ATTCCTGGCGTTACCTTG	GACTGATCCCATTGATTTCC
Mtor/mTOR	TGGCAAGAGCGGCAGACTG	GTTGGCAGAGGATGGTCAAGTTG
